# Supplementary material for: Procalcitonin for antimicrobial stewardship among cancer patients admitted with COVID-19
Source: eLife. 2022 Dec 21;11:e81151. doi: 10.7554/eLife.81151 (PMC9788806; doi:10.7554/eLife.81151)
Supplement: Supplementary file 1. [file elife-81151-supp1.docx]

Supplementary file 1. Independent impact of PCT on outcomes by multivariable logistic regression analysis.

| Outcome | Independent predictor | aOR | 95% CI | *P*-value |
| --- | --- | --- | --- | --- |
|  |  |  |  |  |
|  |  |  |  |  |
| **Positive bacterial culture** | PCT ≥ 0.25 ng/ml | 3.80 | 2.07 to 6.97 | < .0001 |
|  | Gender |  |  | 0.01 |
|  | Male | Reference |  |  |
|  | Female | 2.23 | 1.20 to 4.14 |  |
|  |  |  |  |  |
| **Use of IV antibiotics** | PCT ≥ 0.25 ng/ml | 2.36 | 1.30 to 4.28 | 0.005 |
|  | Type of cancer |  |  | 0.002 |
|  | Solid tumor | Reference |  |  |
|  | Hematological malignancy | 2.82 | 1.59 to 5.00 |  |
|  | Both of above | 1.21 | 0.44 to 3.35 |  |
|  | Obstructive sleep apnea | 2.58 | 1.07 to 6.21 | 0.034 |
|  | ALC < 1000/µl at admission | 1.80 | 1.08 to 3.00 | 0.024 |
|  | Pneumonia | 2.49 | 1.46 to 4.26 | < .001 |
|  | Oxygen supplementation within | 2.99 | 1.78 to 5.04 | < .0001 |
|  | 72 hours |  |  |  |
|  |  |  |  |  |
| **Use of IV antibiotics ≥ 72 hours** | PCT ≥ 0.25 ng/ml | 2.39 | 1.56 to 3.66 | < .0001 |
|  | Type of cancer |  |  | 0.004 |
|  | Solid tumor | Reference |  |  |
|  | Hematological malignancy | 1.98 | 1.31 to 3.00 |  |
|  | Both of above | 1.97 | 0.84 to 4.59 |  |
|  | Obesity | 2.02 | 1.05 to 3.88 | 0.035 |
|  | ALC < 1000/µl at admission | 1.54 | 1.004 to 2.35 | 0.048 |
|  | Oxygen supplementation within | 3.52 | 2.35 to 5.27 | < .0001 |
|  | 72 hours |  |  |  |
|  |  |  |  |  |
| **Use of IV antibiotics ≥ 7 days** | PCT ≥ 0.25 ng/ml | 2.41 | 1.52 to 3.81 | < .001 |
|  | Type of cancer |  |  | < .0001 |
|  | Solid tumor | Reference |  |  |
|  | Hematological malignancy | 2.13 | 1.32 to 3.44 |  |
|  | Both of above | 5.15 | 2.23 to 11.87 |  |
|  | Oxygen supplementation within | 3.94 | 2.28 to 6.80 | < .0001 |
|  | 72 hours |  |  |  |
|  |  |  |  |  |
| **ICU admission** | PCT ≥ 0.25 ng/ml | 2.11 | 1.31 to 3.42 | 0.002 |
|  | Obesity | 2.48 | 1.30 to 4.75 | 0.006 |
|  | Pneumonia | 3.93 | 1.59 to 9.69 | 0.003 |
|  | Oxygen supplementation within | 4.10 | 2.17 to 7.74 | < .0001 |
|  | 72 hours |  |  |  |
|  |  |  |  |  |
| **30-Day mortality** | PCT ≥ 0.25 ng/ml | 3.77 | 2.00 to 7.09 | < .0001 |
|  | Status of cancer |  |  | 0.03 |
|  | Active | 5.14 | 1.17 to 22.56 |  |
|  | No evidence of disease | Reference |  |  |
|  | Coronary artery disease | 4.58 | 1.26 to 16.69 | 0.021 |
|  | Hypertension | 0.41 | 0.20 to 0.84 | 0.014 |
|  | Pneumonia | 3.70 | 1.21 to 11.31 | 0.022 |
|  | Oxygen supplementation within | 3.03 | 1.40 to 6.58 | 0.005 |
|  | 72 hours |  |  |  |

Abbreviation: aOR=Adjusted odds ratio; 95% CI= 95% Confidence interval.
