# Supplementary material for: Procalcitonin for antimicrobial stewardship among cancer patients admitted with COVID-19
Source: eLife. 2022 Dec 21;11:e81151. doi: 10.7554/eLife.81151 (PMC9788806; doi:10.7554/eLife.81151)
Supplement: Supplementary file 2. [file elife-81151-supp2.docx]

| Supplementary file 2. Comparing outcomes between hospitalized COVID-19 patients with different PCT | | | |
| --- | --- | --- | --- |
| values under active cancer treatment. |  |  |  |

| a) ANC < 1000/µl at admission |  |  |  |
| --- | --- | --- | --- |
| Outcomes | PCT < 0.25 ng/ml | PCT ≥ 0.25 ng/ml | *P*-value |
|  | (n=33) | (n=16) |  |
|  | N (%) | N (%) |  |
| Positive bacterial culture | 0 (0) | 1 (6) | 0.33 |
| Duration of hospital stay, median (IQR), days | 7 (5-14) | 5 (4-18) | 0.50 |
| Use of IV antibiotics | 28 (85) | 15 (94) | 0.65 |
| Use of IV antibiotics ≥ 72 hours | 19 (58) | 11 (69) | 0.45 |
| Use of IV antibiotics ≥ 7 days | 6 (18) | 5 (31) | 0.47 |
| ICU admission | 5 (15) | 3 (19) | > .99 |
| Death within 30 days after COVID-19 diagnosis | 2 (6) | 3 (19) | 0.31 |
| b) Under active cancer treatment |  |  |  |
| Outcomes | PCT < 0.25 ng/ml | PCT ≥ 0.25 ng/ml | *P*-value |
|  | (n=118) | (n=58) |  |
|  | N (%) | N (%) |  |
| Positive bacterial culture | 4 (3) | 11 (19) | 0.001 |
| Duration of hospital stay, median (IQR), days | 6 (4-10) | 9 (4-15) | 0.052 |
| Use of IV antibiotics | 94 (80) | 55 (95) | 0.009 |
| Use of IV antibiotics ≥ 72 hours | 57 (48) | 40 (69) | 0.01 |
| Use of IV antibiotics ≥ 7 days | 20 (17) | 21 (36) | 0.005 |
| ICU admission | 13 (11) | 17 (29) | 0.002 |
| Death within 30 days after COVID-19 diagnosis | 6 (5) | 9 (16) | 0.04 |
| Abbreviation: ANC= Absolute neutrophil count; IQR=Interquartile range. | |  |  |
| Note: Values in table are number of patients (percentage) unless otherwise indicated. | | |  |
